# A comparison of substrate oxidation during prolonged exercise in men at terrestrial altitude and normobaric normoxia following the coingestion of ^13^C glucose and ^13^C fructose

**DOI:** 10.14814/phy2.13101

**Published:** 2017-01-13

**Authors:** John P. O'Hara, David R. Woods, Adrian Mellor, Christopher Boos, Liam Gallagher, Costas Tsakirides, Nicola C. Arjomandkhah, David A. Holdsworth, Carlton B. Cooke, Douglas J. Morrison, Thomas Preston, Roderick FGJ King

**Affiliations:** ^1^ Research Institute for Sport, Physical Activity and LeisureLeeds Beckett UniversityLeedsUnited Kingdom; ^2^ Royal Centre for Defence MedicineBirminghamUnited Kingdom; ^3^ Northumbria NHS Trust and Newcastle TrustNewcastleUnited Kingdom; ^4^ James Cook University HospitalMiddlesboroughUnited Kingdom; ^5^ Department of CardiologyPoole HospitalPooleDorsetUnited Kingdom; ^6^ School of Social and Health SciencesLeeds Trinity UniversityLeedsUnited Kingdom; ^7^ Scottish Universities Environmental Research CentreGlasgowUnited Kingdom

**Keywords:** Altitude, carbon isotope, exogenous carbohydrate oxidation, liver glycogen, muscle glycogen, plasma glucose oxidation

## Abstract

This study compared the effects of coingesting glucose and fructose on exogenous and endogenous substrate oxidation during prolonged exercise at altitude and sea level, in men. Seven male British military personnel completed two bouts of cycling at the same relative workload (55% *W*_max_) for 120 min on acute exposure to altitude (3375 m) and at sea level (~113 m). In each trial, participants ingested 1.2 g·min^−1^ of glucose (enriched with ^13^C glucose) and 0.6 g·min^−1^ of fructose (enriched with ^13^C fructose) directly before and every 15 min during exercise. Indirect calorimetry and isotope ratio mass spectrometry were used to calculate fat oxidation, total and exogenous carbohydrate oxidation, plasma glucose oxidation, and endogenous glucose oxidation derived from liver and muscle glycogen. Total carbohydrate oxidation during the exercise period was lower at altitude (157.7 ± 56.3 g) than sea level (286.5 ± 56.2 g, *P* = 0.006, ES = 2.28), whereas fat oxidation was higher at altitude (75.5 ± 26.8 g) than sea level (42.5 ± 21.3 g, *P* = 0.024, ES = 1.23). Peak exogenous carbohydrate oxidation was lower at altitude (1.13 ± 0.2 g·min^−1^) than sea level (1.42 ± 0.16 g·min^−1^, *P* = 0.034, ES = 1.33). There were no differences in rates, or absolute and relative contributions of plasma or liver glucose oxidation between conditions during the second hour of exercise. However, absolute and relative contributions of muscle glycogen during the second hour were lower at altitude (29.3 ± 28.9 g, 16.6 ± 15.2%) than sea level (78.7 ± 5.2 g (*P* = 0.008, ES = 1.71), 37.7 ± 13.0% (*P* = 0.016, ES = 1.45). Acute exposure to altitude reduces the reliance on muscle glycogen and increases fat oxidation during prolonged cycling in men compared with sea level.

## Introduction

High altitude exposure decreases the partial pressure of arterial oxygen, thus reducing arterial oxygen saturation, known as hypoxic hypoxemia. This reduction in oxygen delivery as a combination of reduced oxygen binding to hemoglobin and alteration in blood flow is likely to have a direct effect on fuel use at rest and especially during exercise. It has been assumed that during exercise at altitude there would be a higher contribution of carbohydrate to the total energy yield because free fatty acid oxidation requires more oxygen per ATP molecule synthesized than the complete oxidation of carbohydrate (Hinkle et al. 1991).

There are limited studies that have investigated whole body substrate oxidation during prolonged exercise in acute hypoxia, with equivocal findings. Friedmann et al. (2004) and Katayama et al. (2010) showed the respiratory exchange ratio (RER) to be higher during acute exposure to hypoxia compared with normoxia at high (~80% of altitude‐related maximal oxygen uptake ($\dot{V}O_{2\max}$)) and moderate (50% of altitude‐related peak oxygen uptake) relative exercise intensities, respectively. In contrast, other studies have shown no change in RER in acute exposure to hypoxia compared with normoxia at similar relative exercise intensities (Lundby and Van Hall 2002; Bouissou et al. 1987; Young et al. 1982). Conversely, Lundby and Van Hall (2002) showed greater carbohydrate oxidation when the same absolute exercise intensity was used. However, such mixed gender studies may be difficult to interpret since females have been shown to have lower whole body carbohydrate oxidation at altitude (Beidleman et al. 2002), although this was not supported by Lundby and Van Hall (2002).

To the best of our knowledge, there have only been two studies which have evaluated the effects of an exogenous source of carbohydrate in acute hypoxia on substrate oxidation (Peronnet et al. 2006) and exercise performance (Fulco et al. 2005). Fulco et al. (2005) established that despite hypoxia (4300 m) the ingestion of a 10% carbohydrate solution enhanced time trial performance by ~7% compared with a placebo (though still not equivalent to a sea level performance). This was attributed to greater availability of plasma glucose, but this study was unable to confirm the effect of ingesting carbohydrate on fuel selection.

Only one study that the authors are aware of has investigated fuel use in acute exposure to hypoxia (4300 m) using ^13^C tracer techniques (Peronnet et al. 2006). The relative contribution of exogenous carbohydrate oxidation to the total energy yield following the ingestion of 140 g of glucose was not significantly higher in hypoxia (14.9 ± 1.1%) compared with normoxia (12.5 ± 1.5%) during the last 40 min of a total of 80 min of cycling at ~77% of altitude specific $\dot{V}O_{2\max}$ (169 ± 10 W and 234 ± 10 W, respectively). Neither was it higher compared with the same absolute workload (169 ± 10 W) in normoxia (16.8 ± 1.1%). This was despite higher plasma glucose concentrations during hypoxia, and regardless of evidence showing that there is a greater dependency on plasma glucose during exercise in acute hypoxia compared with normoxia when using the same absolute exercise intensity (Roberts et al. 1996b; Brooks et al. 1991a,b). However, whether the outcome would have been different, following the ingestion of multiple transportable carbohydrates (e.g., glucose and fructose), which have been shown to produce higher exogenous oxidation rates in normoxia compared with glucose alone (Jentjens and Jeukendrup 2005; Adopo et al. 1994), is yet to be established. In addition, Peronnet et al. (2006) showed the relative contribution of whole body carbohydrate oxidation to the total energy yield to be significantly higher in hypoxia (92 ± 2.1%) than in normoxia for both a given absolute (75.3 ± 5.2%) and relative workload (78.1 ± 1.8%). This was attributed to hypoxia placing a greater reliance on endogenous glucose oxidation, yet, this study did not distinguish between the relative contributions of liver and muscle glycogen utilization. This warrants investigation with a particular focus on the contribution of the liver, as muscle glycogen depletion has been shown to be *largely* lower following acute hypoxia compared with normoxia (Young et al. 1982), *albeit* only for 30 min of exercise. Therefore, the purpose of the present study was to compare the effects of coingesting glucose and fructose during 120 min of moderate intensity cycling exercise on fuel use during acute hypoxia (terrestrial altitude) and normobaric normoxia (sea level) in men. The use of indirect calorimetry combined with ^13^C tracer techniques enabled the estimation of exogenous and endogenous (liver and muscle) contributions to carbohydrate oxidation.

## Methods

### Participants

Seven male British military personnel, aged: 27 ± 5 years, with a body mass of 77.7 ± 5.9 kg, were recruited for this study, all of whom were engaged in regular physical training (3–5 training days per week). Participants were considered to be physically fit but not elite athletes. Procedures and potential risks were explained and each participant provided written informed consent prior to the study. The study was approved by the Ministry of Defence Research Ethics Committee and was conducted in accordance with the declaration of Helsinki.

### Preliminary testing

Participants completed two maximal incremental cycle tests to volitional exhaustion to determine their individual maximal workload (*W*_max_ (Kuipers et al. 1987)) and $\dot{V}O_{2\max}$ on a bicycle affixed to a bicycle trainer (Compu Trainer^®^ Pro Lab, Race Mate Inc, Seattle, WA, USA). The bicycle trainer was calibrated following the manufacturer's instructions. The first test was performed at sea level (absolute altitude ~113 m) with the second test performed a week later during acute exposure to normobaric hypoxia (an FiO_2_ of ~13.4% (considering water vapor partial pressure [Conkin 2011; Fenn et al. 1946] and daily fluctuations of barometric pressure) equivalent to 3375 m (the reported altitude for the New Refuge Torino in the Italian Alps; PiO_2_ 95.2 mmHg) in an environmental chamber (TISS, Alton, UK and Sporting Edge, Sherfield on Loddon, UK). Oxygen uptake ($\dot{V}O_{2}$) and carbon dioxide production ($\dot{V}{\mathrm{CO}}_{2}$) measurements were made throughout the test using an online gas analysis system (Metalyser, Cortex, Germany), which was calibrated following the manufacturer's instructions. $\dot{V}O_{2}$ and $\dot{V}{\mathrm{CO}}_{2}$ were calculated by the online gas analysis system using standard metabolic algorithms (Wasserman et al. 1999) using the Haldane transformation. FiO_2_ and FiCO_2_ were measured continuously, rather than assuming constants, thus correcting for changes in ambient conditions. This is of particular important in a normobaric chamber where the FiO_2_ is reduced to simulate a given altitude. At sea level and normobaric hypoxia, participants achieved *W*_max_ values of 239.8 ± 20.7 W and 209.0 ± 17.6 W and $\dot{V}O_{2\max}$ values of 48.4 ± 4.9 mL·kg^−1^·min^−1^ and 40.2 ± 4.9 mL·kg^−1^·min^−1^, respectively.

### Design of the study

Following the assessment of maximal workloads, participants completed two experimental cycle trials for 120 min at 55% *W*_max._ The first was at terrestrial altitude (barometric pressure 506.7 ± 1.4 mmHg, PiO_2:_ 96.3 ± 0.3 mmHg [New Refuge Torino, Alps, Italy]). The altitude trial involved rapid ascent (30 min) by a cable car to the New Refuge Torino. This took place 24–72 h after the participants had been driven from sea level to <1500 m where they rested prior to ascent. Thereafter, the second assessment at sea level was made 7 weeks later. Each cycling test involved the ingestion of 1.8 g·min^−1^ of carbohydrate (1.2 g·min^−1^ of glucose [d‐glucose, Thornton and Ross Ltd, Huddersfield, UK] and 0.6 g·min^−1^ of fructose [d‐fructose, Danisco, Oy, Oktka, Finland]) at regular intervals during exercise. Each 2 L formulation contained 26 mmol L^−1^ sodium chloride and flavoring. Stock glucose (natural *δ* ^13^C abundance = −32.58‰) and fructose (natural δ^13^C abundance = −30.04 ‰), was enriched using 0.24 g of U‐^13^C_6_ d‐glucose (Cambridge Isotope Laboratories, Inc, Tewksbury, MA, USA), and 0.12 g of U‐^13^C_6_ d‐fructose (Cambridge Isotope Laboratories, Inc), achieving a final enrichment of δ^13^C = +115.88 ‰. All δ^13^C measurements are quoted with reference to the internationally accepted standard for carbon isotope measurements, Vienna Pee Dee Belemnite (VPDB). The ^13^C abundance of stock glucose and fructose and ^13^C enrichment of spiked glucose and fructose was determined using liquid chromatography coupled to isotope ratio mass spectrometry (LC–IRMS; Isoprime, Cheadle, UK), using l‐fucose as an isotopic internal standard as previously described (Morrison et al. 2011).

### Diet and physical activity before testing

Participants recorded their food intake and activity patterns during the 72‐h prior to the first experimental trial and were instructed to repeat the same diet and activity pattern in the 72‐h before the subsequent trial. Participants were required to refrain from any intense and/or prolonged physical activity, alcohol, or caffeine consumption in the 36‐h prior to each experimental trial. In addition, they were asked to refrain from ingesting carbohydrates derived from plants which utilize the C_4_ photosynthetic cycle, in which there is higher natural abundance of ^13^C (e.g., maize‐derived sugars (Morrison et al. 2000)) for the duration of the study. This precaution ensured that background ^13^CO_2_ abundance was less likely to be perturbed from oxidation of endogenous and dietary substrate stores from naturally “enriched” C4 origin. A standardized evening meal was consumed 12 h prior to each experiment trial (total 1443 kcal; 53% carbohydrate, 17% fat, 30% protein).

### Experimental trials

Each experimental trial was performed at 19–21°C. Participants repeated their trials at the same time of day, although this varied between 9 am and 2 pm for individuals, to avoid any influence of circadian variance, following a 12‐h fast. On arrival, a catheter (20 gauge Introcan Safety^®^, B. Braun Medical Ltd, Sheffield, UK) was inserted into an antecubital vein for regular blood sampling. After 20 min of acute exposure to each environmental condition, peripheral oxygen saturation (SpO_2_; Nellcor N‐20, Covidien, Dublin, Ireland) was measured and resting blood samples were drawn for the analysis of plasma glucose, serum insulin, serum free fatty acids, plasma lactate, and plasma glucose ^13^C enrichment concentrations, as well as plasma metanephrine and normetanephrine.

Over the next 10 min resting, $\dot{V}O_{2}$ and $\dot{V}{\mathrm{CO}}_{2}$ measurements were made using an online gas analysis system (Metalyser, Cortex, Germany), which was calibrated following the manufacturer's instructions. For the measurement of ^13^C/^12^C in expired CO_2_, 12 mL samples of expired gas were collected in duplicate in Labco Exetainers^®^ (SerCon Ltd, Crewe, UK) via a mixing chamber (Jaeger, Germany).

After a 5 min standardized warm up, which included the calibration of the bicycle trainer (Compu Trainer Pro Lab, Racer Mate Inc, USA) to the manufacturer's instructions, an initial bolus of the carbohydrate solution was consumed (397 mL). Participants then completed 120 min of cycling; 5 min at 40% *W*_max_, 5 min at 45% *W*_max_, 5 min at 50% *W*_max_, and 105 min at 55% *W*_max_. These workloads were calculated from each individual's sea level and normobaric hypoxic *W*_max_ for the sea level and altitude environments, respectively. Additional boluses (229 mL) of the carbohydrate solution were provided every 15 min throughout the 120 min exercise period. Expired gas breath samples were collected and measurements of $\dot{V}O_{2}$ and $\dot{V}{\mathrm{CO}}_{2}$ were made every 15 min during exercise until the end of exercise. Samples of expired gas for ^13^C CO_2_ analysis were collected during the final 60 sec of each expired gas collection period. Samples for the analysis of plasma glucose, serum insulin, serum free fatty acids, and plasma lactate were drawn every 15 min, those for ^13^C plasma glucose enrichment were drawn at 60, 90, and 120 min, and those for plasma metanephrine and normetanephrine concentrations were drawn at 60 and 120 min. Heart rate, rating of perceived exertion (RPE (Borg 1982)), and SpO_2_ was measured every 15 min during cycling exercise.

### Analyses

Aliquots of plasma and serum, which were collected during both conditions, were prepared by centrifugation. The samples collected at altitude, were stored temporarily at −20°C until they were transported back to the United Kingdom, where they were then stored at −80°C until analysis, as per the samples collected at sea level. The stored plasma and serum was subsequently analyzed for selected metabolites. Plasma glucose (Glucose Oxidase kit, Instrumentation Laboratory (ILab), Monza, Itally), plasma lactate (Lactate kit, Randox, County Antrim, UK), and serum free fatty acid (NEFA‐HR2, Wako Chemicals GmbH, Germany) were analyzed enzymatically (ILab 300 plus, ILab, UK). Serum insulin was analyzed using an antibody assay (Insulin IRI kit, Siemens Healthcare Diagnostics Inc, Camberley, UK) using a semiautomatic analyzer (ADVIA Centaur^®^ System, Bayer Diagnostics, Newbury, Berks, UK). The within‐run precision (coefficient of variation) for plasma glucose, plasma lactate, serum insulin and serum free fatty acid was 2.6%, 2.9%, 1.5%, and >5.9%, respectively. Plasma metanephrine and normetanephrine assays was performed by LC–MSMS using a tandem quadrupole mass spectrometer (Waters UK, Hertfordshire, UK) with positive electrospray ionization. As well as the sample, a quality control and internal standard are used for quantitation. The lower limit of quantification is between 40 and 50 pmol/L with a CV of 13–16%.

The ^13^C/^12^C ratio in expired CO_2_ was determined using isotope ratio mass spectrometry (IRMS; AP2003, GVI Instruments Ltd, Manchester, UK). The isotopic ratio ^13^C/^12^C is derived against laboratory CO_2_ (itself calibrated against VPDB) from the ion beam area ratio measurements with correction of the small contribution of ^12^C^16^O^17^O at m/z 45, the Craig correction (Craig 1957). The ^13^C/^12^C ratio in plasma glucose was determined using LC–IRMS as described in detail previously (Morrison et al. 2011).

Oxidation rates of total fat, total carbohydrate, endogenous carbohydrate (liver and muscle), plasma glucose, and exogenous carbohydrate derived from glucose and fructose ingestion combined, were calculated by indirect calorimetry ($\dot{V}O_{2}$ and $\dot{V}{\mathrm{CO}}_{2}$) and stable isotope measurements (^13^C/^12^C ratio in expired CO_2_ and plasma glucose), as detailed below.

### Calculations

Total carbohydrate and fat oxidation (g·min^−1^) were computed from $\dot{V}O_{2}$ (L·min^−1^) and $\dot{V}{\mathrm{CO}}_{2}$ (L·min^−1^) using stoichiometric equations (Peronnet and Massicotte 1991), with the assumption that protein oxidation during exercise was negligible.

(1) $$\mathrm{Carbohydrate}\left(g\cdot \min^{-1}\right)=4.585\dot{V}{\mathrm{CO}}_{2}-3.226\dot{V}O_{2}$$

(2) $$\mathrm{Fat}\left(g\cdot \min^{-1}\right)=1.695\dot{V}O_{2}-1.701\dot{V}{\mathrm{CO}}_{2}$$

The isotopic enrichment of the ingested glucose and fructose (R_exo_), was expressed in standard δ^13^C units (‰) relative to VPDB (Craig 1953). The rate of exogenous carbohydrate oxidation derived from the combined ingestion of glucose and fructose was computed using the following equation (Mosora et al. 1981).

(3) $$\mathrm{Exogenous}\;\mathrm{Carbohydrate}\;\mathrm{Oxidation}\left(g\cdot \min^{-1}\right)=\dot{V}{\mathrm{CO}}_{2}[R_{\exp}-R_{\mathrm{ref}}/\left(R_{\mathrm{exo}}-R_{\mathrm{ref}}\right]/k$$

where $\dot{V}{\mathrm{CO}}_{2}$ is in liters per min, R_exp_ is the measured isotopic composition in expired CO_2_, R_ref_ is the isotopic composition of expired CO_2_ at rest prior to exercise and carbohydrate ingestion, R_exo_ is the measured isotopic composition of the exogenous glucose and fructose ingested, and *k* (0.7426 L·g^−1^) is the rate adjusted value for the complete oxidation of glucose (Peronnet et al. 1990). Endogenous carbohydrate oxidation was calculated by subtracting exogenous carbohydrate oxidation from total carbohydrate oxidation.

Computations were made on the assumption that, in response to exercise, ^13^C is not irreversibly lost in pools of tricarboxylic acid cycle intermediates and/or bicarbonate, and that ^13^CO_2_ recovery in expired gases was complete or almost complete during exercise (Trimmer et al. 2001). Such computation has been shown to underestimate exogenous carbohydrate oxidation rates at the beginning of exercise because of the delay between ^13^CO_2_ production in tissues and expired ^13^CO_2_ at the mouth (Pallikarakis et al. 1991). Based on this, exogenous carbohydrate oxidation rates are presented from 60 min onwards during the exercise period, where it is expected that there would be isotopic equilibrium in the tissues and at the mouth.

On the basis of the isotopic compositions of plasma glucose (R_glu_), the oxidation rate of plasma glucose was computed at 60, 90, and 120 min during exercise (Peronnet et al. 1998).

(4) $$\mathrm{Plasma}\;\mathrm{glucose}\;\mathrm{oxidation}\left(g\cdot \min^{-1}\right)=\dot{V}{\mathrm{CO}}_{2}\left[\left(R_{\exp}-R_{\mathrm{ref}}\right)/\left(R_{\mathrm{glu}}-R_{\mathrm{ref}}\right)\right]/k$$

The oxidation rate of muscle glycogen (g·min^−1^), either directly or through the lactate shuttle (Brooks 1986), was calculated by subtracting plasma glucose oxidation (eq.(4)) from total carbohydrate oxidation (eq. (2)). Finally, the amount of glucose released from the liver was estimated as the difference between plasma glucose (eq. (4)) and exogenous carbohydrate oxidation (eq. (4)) (Peronnet et al. 1998).

### Statistical analysis

Data were normally distributed (Kolmogorov–Smirnov test) and are presented as mean ± SD. Graphs were drawn using GraphPad Prism 7 (GraphPad Software, Inc., La Jolla, CA). Two‐way ANOVA for repeated measures was used to compare differences in fuel use and blood‐related variables over time and between conditions. Where significance was detected post hoc analysis was performed between the two conditions at specific time points using a paired *t*‐test. Paired *t*‐tests were used to compare differences in relative and absolute fuel use, as well as heart rate, SpO_2_ and RPE between conditions. This was supported with 95% confidence intervals. Data were evaluated using SPSS for Windows version 22 (Chicago, Illinois, USA). A 0.95 level of confidence was predetermined to denote statistical significance (*P* < 0.05). Cohen's *d* effect sizes were calculated, and interpreted using a modified effect size (ES) scale, where 0–0.2 was considered to be a *trivial* effect, 0.2–0.6 a *small* effect, 0.6–1.2 a *moderate* effect, 1.2–2.0 a *large* effect, and >2.0 a *very large* effect (Batterham and Hopkins 2006).

## Results

### Total carbohydrate and fat oxidation

**Figure 1:**
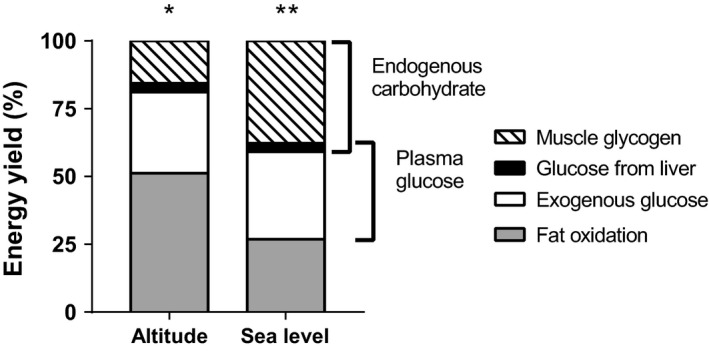
The relative (% of energy yield) contribution of exogenous and endogenous substrate oxidation during the second hour of cycling at altitude and sea level. *fat oxidation significantly higher at altitude than sea level (*P* = 0.023). **muscle glycogen oxidation significantly higher at sea level than altitude (*P* = 0.016).

The total energy expenditure for 2 h of continuous cycling was significantly lower at altitude (1346.55 ± 123.7 kcal) compared with sea level (1522.74 ± 123.8 kcal, *P* = 0.005, ES = 1.42). Absolute carbohydrate oxidation was significantly lower at altitude compared with sea level with a *very large, large,* and *very large* effect during the first hour, second hour, and for the entire two of hours of continuous cycling, respectively (Table 1). In contrast, absolute fat oxidation was significantly higher at altitude compared with sea level with a *large*,* moderate, and large* effect during the first hour, second hour, and for the 2 h of exercise, respectively (Table 1). In addition, the relative contribution of fat oxidation to the total energy yield was also significantly higher at altitude (51.2 ± 18.9%, *P* = 0.023) compared with sea level (26.9 ± 13.1%), with a *large* effect (ES = 1.28) for the second hour of exercise (Fig. 1).

**Table 1:** Comparisons of total carbohydrate and fat oxidation for the 2 h of exercise, as well as for the initial and second hour of exercise

	Total oxidation (g)	Difference in total oxidation (g) Altitude vs. sea level
Carbohydrate 2 h of exercise		
Sea level	286.47 ± 56.23	
Altitude	157.72 ± 56.34	−128.74, −204.00 to −53.49 ES = 2.28, *P* = 0.006
First hour		
Sea level	127.79 ± 27.80	
Altitude	69.31 ± 20.80	−58.48, −88.91 to −28.04 ES = 2.81, *P* = 0.003
Second hour		
Sea level	158.68 ± 29.79	
Altitude	88.41 ± 37.10	−70.27, −116.45 to −24.08 ES = 1.89, *P* = 0.01
Fat *2 *h of exercise		
Sea level	42.47 ± 21.33	
Altitude	75.51 ± 26.80	33.03, 6.18–59.89 ES = 1.23, *P* = 0.024
First hour		
Sea level	19.49 ± 10.09	
Altitude	37.47 ± 12.81	17.97, 4.99–30.95 ES = 1.40, *P* = 0.015
Second hour		
Sea level	22.98 ± 11.30	
Altitude	38.04 ± 14.87	15.06, 0.29–29.82 ES = 1.01, *P* = 0.047

Absolute values (1st line: means SD and differences among trials with the associated 95% confidence limits of the difference; 2nd line: Cohen's ES and *P* values [Paired *t*‐test]).

### δ ^13^CO_2_ in expired gas and δ ^13^C in plasma glucose

**Figure 2:**
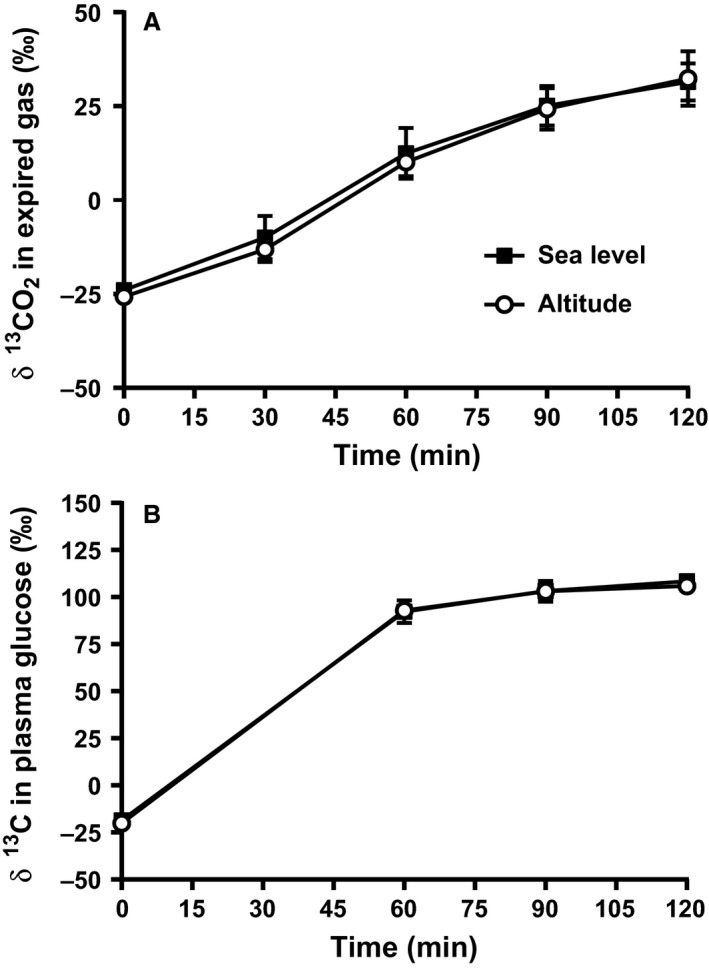
Changes in δ ^13^CO_2_ in expired CO_2_ (A) and δ ^13^C in plasma glucose (B) at rest and during the 2 h of cycling at altitude and sea level.

The δ ^13^CO_2_ in expired gas (−24.0 ± 1.3 vs. −25.8 ± 0.9‰) and δ ^13^C in plasma glucose (−18.6 ± 1.7 vs. −20.1 ± 1.9%) were similar at sea level and altitude at rest before exercise and coingestion of ^13^C glucose and ^13^C fructose. Both significantly increased over time (main effect: *P* < 0.001) from the start of exercise following the coingestion of ^13^C glucose and ^13^C fructose (Fig 2A and B). The δ ^13^CO_2_ in expired gas (31.4 ± 4.9 vs. 29.7 ± 8.8%) and δ ^13^C in plasma glucose (108.5 ± 1.9 vs. 106.0 ± 3.5%) were maximal at 120 min for sea level and altitude (Fig. 2A and B). There were no significant differences between conditions during exercise, with effect sizes being *trivial* to *moderate* for both δ ^13^CO_2_ in expired gas (ES range = 0.13–0.96) and δ^13^C in plasma glucose (ES range = 0.04–0.73).

### Exogenous and endogenous carbohydrate oxidation

**Figure 3:**
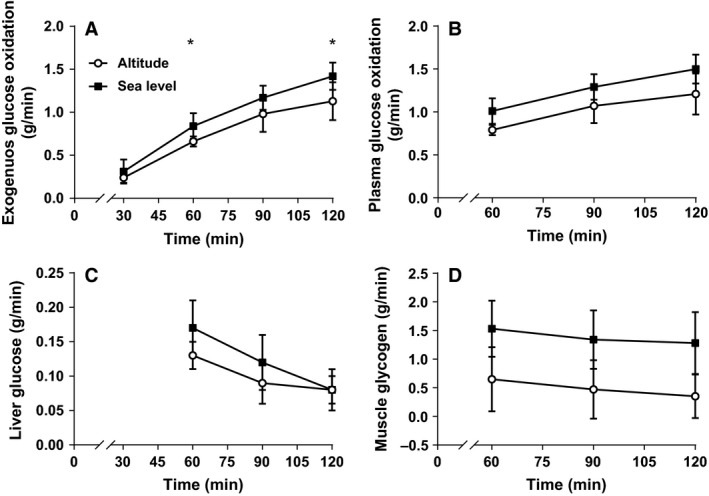
Oxidation rates of exogenous carbohydrate (A), plasma glucose (B), glucose released from the liver (C), and muscle glycogen (D) during the second hour of cycling. *sea level significantly higher than altitude (*P* < 0.05).

Exogenous carbohydrate oxidation was significantly lower at 60 min at altitude (0.66 ± 0.06 g·min^−1^, *P* = 0.036) compared with sea level (0.84 ± 0.15 g·min^−1^), with a *very large* effect size (ES = 3.08), Figure 3A. Furthermore, exogenous carbohydrate oxidation rates were maximal at 120 min of exercise, being significantly lower at altitude (1.13 ± 0.22 g·min^−1^, *P* = 0.034) than sea level (1.42 ± 0.16 g·min^−1^), with a *large* effect size (ES = 1.33). However, the relative contribution of exogenous carbohydrate oxidation to the total energy yield for altitude (29.9 ± 4.4%) and sea level (32.2 ± 3.3%), was similar between conditions, with a *small* effect size (ES = 0.52), Figure 1. In addition, there was a *moderate* effect for absolute exogenous carbohydrate oxidation during the second hour of exercise (Table 2), although not significantly different between altitude and sea level. Table 2 also shows that there was a *large* and significant reduction in the reliance on absolute endogenous carbohydrate oxidation at altitude compared with sea level for the second hour of exercise.

**Table 2:** Comparisons of carbohydrate oxidation from various sources between acute altitude exposure and sea level conditions, over the second hour of exercise

	Carbohydrate oxidation (g)	Difference in carbohydrate oxidation (g) Altitude vs. sea level
Exogenous glucose		
Sea level	67.85 ± 8.23	
Altitude	56.21 ± 10.20	−11.64, −26.67 to 3.38 ES = 1.14, *P* = 0.107
Endogenous carbohydrate		
Sea level	90.27 ± 28.43	
Altitude	34.32 ± 30.24	−55.95, −93.13 to −18.77 ES = 1.85, *P* = 0.01
Muscle glycogen		
Sea level	78.74 ± 5.21	
Altitude	29.32 ± 28.94	−49.43, −80.27 to −18.58 ES = 1.71, *P* = 0.008
Glucose from liver		
Sea level	6.84 ± 2.28	
Altitude	5.94 ± 1.32	−0.90, −3.22 to 1.44 ES = 0.68, *P* = 0.383
Plasma glucose		
Sea level	74.69 ± 9.08	
Altitude	62.16 ± 10.29	−12.54, −27.53 to 2.45 ES = 1.22, *P* = 0.087

Absolute values (1st line: means SD and differences among trials with the associated 95% confidence limits of the difference; 2nd line: Cohen's ES and *P* values [Paired *t*‐test]).

### Oxidation of plasma glucose, liver glucose and muscle glycogen

Figure 3B shows that there were no significant differences in the rate of plasma glucose oxidation between altitude and sea level during the second hour of exercise. However, the lower rates at altitude compared with sea level produced *very large, moderate, and large* effect sizes at 60 (ES = 3.50), 90 (ES = 1.07), and 120 min (ES = 1.25). In addition, the absolute contribution of plasma glucose to the total energy yield was not significantly different between conditions, although there was a *large* effect size (Table 2). There were no significant differences in the rate of liver glucose oxidation between conditions (Fig. 3C). The lower rates at altitude produced *large, moderate,* and *trivial* effect sizes at 60 (ES = 2.02), 90 (ES = 0.80), and 120 min (ES = 0.12), respectively. There were also no significant differences in the relative (altitude: 3.2 ± 0.9% vs. sea level: 3.2 ± 0.9%, ES = 0.03, Fig. 1) and absolute (Table 2) contributions of liver glucose to the total energy yield between conditions, with *trivial* and *moderate* effect sizes, respectively. The rate of muscle glycogen oxidation shown in Figure 3D is lower at altitude than sea level, during the second hour of exercise, with effects sizes ranging from *large* to *very large* (ES range = 1.49–2.26). However, there were no significant differences detected between conditions. In contrast, the relative contribution of muscle glycogen to the total energy yield was significantly lower at altitude (16.6 ± 15.2%, *P* = 0.016) compared with sea level (37.7 ± 13.0%), with a *large* effect size (ES = 1.45) as shown in Figure 1. The absolute contributions of muscle glycogen were also significantly lower at altitude (Table 2), for the second hour of exercise, with a *large* effect size.

### Blood Biochemistry

**Figure 4:**
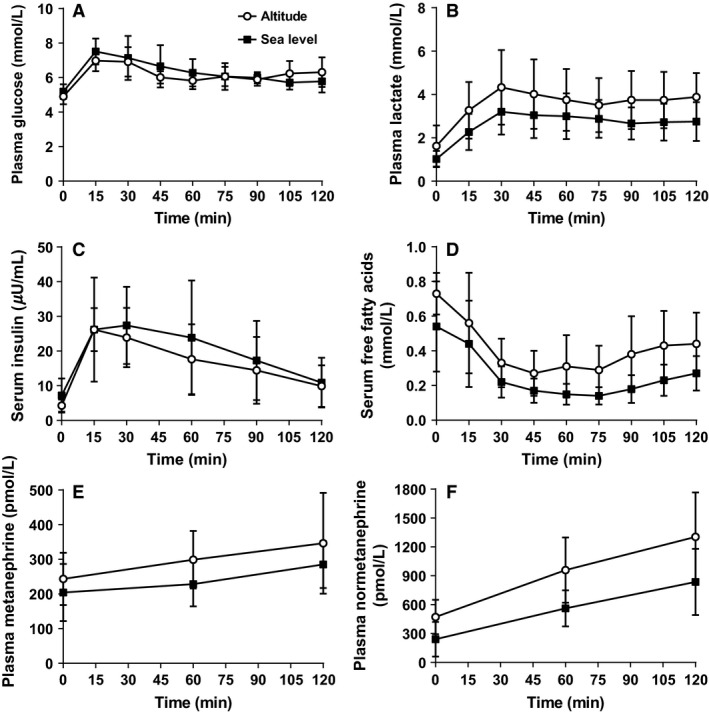
Plasma glucose (A), plasma lactate (B), serum insulin (C), serum free fatty acids (D), plasma metanephrine (E), and plasma normetanephrine (F) concentrations at rest and during 2 h of cycling.

Baseline plasma glucose (4.9 ± 0.5 vs. 5.2 ± 0.6 mmol·L^−1^, ES = 0.63, Fig. 4A), plasma lactate (1.9 ± 1.2 vs. 1.2 ± 0.3 mmol·L^−1^, ES = 0.56, Fig. 4B), serum free fatty acid (0.7 ± 0.1 vs. 0.5 ± 0.3 mmol·L^−1^, ES = 2.28, Fig. 4C), and serum insulin (4.3 ± 1.8 vs. 7.2 ± 4.9 *μ*U·mL^−1^, ES = 0.72, Fig. 4D) concentrations at altitude and sea level were not significantly different at rest before the coingestion of ^13^C glucose and ^13^C fructose. Furthermore, there were no significant differences in resting metanephrine (243.6 ± 75.5 vs. 204.5 ± 84.5 pmol·L^−1^, ES = 0.52, Fig. 4E) and normetanephrine (472.0 ± 178.3 vs. 240.0 ± 180.3 pmoL·l^−1^, ES = 1.30, Fig. 4F) between altitude and sea level.

Following the start of exercise, plasma glucose concentrations increased to 7.0 ± 06 and 7.5 ± 0.8 mmol·L^−1^ for altitude and sea level, respectively. At 30 min, plasma glucose concentrations decreased slightly, then remained stable, but elevated above basal concentrations for both conditions (pooled average for 45–120 min = 6.2 ± 07 mmol·L^−1^). However, there were no significant differences between conditions, with effect sizes ranging from *trivial* to *large* (ES range = 0.0–1.78). Even though plasma lactate concentrations during exercise were higher at altitude compared with sea level (average difference of 0.86 ± 1.3 mmol·L^−1^, ES = 0.62), there were no significant differences in responses between conditions, with *small* to *moderate* effect sizes (ES = 0.47–0.82). Serum free fatty acid concentrations were higher throughout exercise at altitude (average difference of 0.18 ± 0.18 mmol·L^−1^, ES = 0.96) and serum insulin concentrations were lower (average difference of 2.7 ± 9.3 μU·mL^−1^, ES = 0.30), compared with sea level. However, there were no significant differences in responses between conditions for both free fatty acid and insulin concentrations, with *moderate* (ES = 0.74–1.06) and *trivial* to *moderate* effect sizes (ES = 0.0–0.72), respectively. Both metanephrine and normetanephrine concentrations increased during exercise for both conditions and were greater at altitude compared with sea level (average difference of 66.0 ± 87.7 pmol·L^−1^, ES = 0.42–0.85 and 433.8 ± 262.6 pmol·L^−1^, ES = 1.01–1.17, respectively), however, there were no significant differences between conditions.

### Heart rate, rating of perceived exertion, and SpO_2_

There was a *large* difference in the mean heart rate during the 2 h of cycling between conditions, being significantly higher at altitude compared with sea level (*P* = 0.023, ES = 1.28 (Table 3)). Furthermore, there was a *very large* and significant difference during the initial hour (*P* = 0.002, ES = 2.30) for heart rate, but only a *small* insignificant difference during the last hour (ES = 0.50).

**Table 3:** Mean heart rate, rating of perceived exertion, and peripheral oxygen saturation over the 2 h of cycling, as well as for the initial and second hour of exercise

	Altitude	Sea level
2 h of exercise		
HR (beats·min^−1^)	151.28 ± 6.38^a^	143.10 ± 7.67
RPE	13.5 ± 1.76	12.1 ± 3.64
SpO_2_	81.54 ± 3.30^c^	95.45 ± 0.67
First hour		
HR (beats·min^−1^)	148.29 ± 5.22^b^	136.27 ± 5.56
RPE	12.0 ± 1.65	10.6 ± 3.13
SpO_2_	81.6 ± 3.47^c^	95.54 ± 0.85
Second hour		
HR (beats·min^−1^)	155.53 ± 7.82	150.61 ± 10.68
RPE	15.04 ± 2.49	13.5 ± 4.37
SpO_2_	81.46 ± 3.47^c^	95.36 ± 0.66

a Significantly higher than sea level (*P* = 0.023).

b Significantly higher than sea level (*P* = 0.002).

c Significantly lower than sea level (*P* < 0.001).

RPE was *moderately* higher at altitude compared with sea level, during the 2 h of cycling (ES = 0.74), as well as during the initial (ES = 0.80) and second hour (ES = 0.62) of cycling. However, there were no significant differences in RPE between conditions. There were *very large* differences in the mean SpO_2,_ with significantly lower values at altitude for the 2 h of cycling (*P* ≤ 0.001, ES = 4.85), as well as during the initial (*P *≤ 0.001, ES = 4.43) and second hour (*P* ≤ 0.001, ES = 5.17) of cycling (Table 3).

## Discussion

This is, to our knowledge, the first study to compare the effects of coingesting glucose and fructose on exogenous and endogenous (liver and muscle) carbohydrate oxidation, as well as fat oxidation at terrestrial altitude and sea level in males. The primary findings are that despite significant hypoxemia at altitude, whole body carbohydrate oxidation (relative and absolute contributions) was lower and there was a greater reliance on fat oxidation (relative and absolute contributions) compared with sea level. Exogenous carbohydrate oxidation made a significant contribution to the total energy yield for both conditions. However, there were no differences in the absolute and relative contributions of exogenous carbohydrate oxidation to the total energy yield between conditions. The lower whole body carbohydrate oxidation at altitude is therefore explained by the lower endogenous carbohydrate oxidation, primarily due to a reduced dependence on preexisting muscle glycogen (including the lactate shuttle), rather than real differences in glucose released from the liver.

A novel finding of this study was that whole body carbohydrate oxidation was lower at altitude compared with sea level in these male participants and that the shortfall is made up by oxidation of fat. This is in contrast to the existing literature in men where a greater dependency on plasma glucose has been shown (Roberts et al. 1996b; Brooks et al. 1991a,b), as well as increased carbohydrate oxidation (Peronnet et al. 2006) in acute hypoxia compared with sea level, when the same absolute exercise intensity was used. These literatures are consistent, and expected. The sea level literature is clear, that an increased relative exercise intensity, requires a greater contribution from carbohydrate to the total energy yield (Brooks and Mercier 1994). Therefore, as maximal oxygen uptake at altitude is reduced (Dill et al. 1931), a given absolute exercise intensity means a higher relative exercise intensity at altitude, compared with sea level, thus a greater dependency on carbohydrate oxidation. However, when the exercise intensity is normalized to the same relative exercise intensity as per this study, by reducing the absolute exercise intensity at altitude, the literature is divergent and in contrast to this study. For example, when no carbohydrate was ingested, the literature for males shows either an increase (Katayama et al. 2010; Friedmann et al. 2004) or no change in RER (Young et al. 1982; Bouissou et al. 1987) in acute hypoxia compared with normoxia. In addition, a group of males and females has shown no change in the relative contribution of carbohydrate to the total energy yield at altitude (Lundby and Van Hall 2002). The present data are also partially different to Peronnet et al. (2006), who demonstrated a higher relative contribution of carbohydrate to the total energy yield at altitude (hypobaric chamber) compared with sea level following glucose ingestion. This is in contrast to our data; however, the absolute amount of carbohydrate utilized was lower at altitude in their study, which supports our data. The difference is difficult to explain, as both studies used similar relative exercise intensities (~74% vs. ~77% $\dot{V}O_{2\max}$) and participants with a similar training status. Whether it is related to the lower altitude used in this study, and the fact the present study is the first to assess fuel use following carbohydrate ingestion at terrestrial altitude, is yet to be established. The existing research, which normalizes the relative exercise intensity in the design of their studies between sea level and altitude are extremely difficult to reconcile, and as such we add a new perspective to the debate. Therefore, further research is required to establish consistency within the literature, with regard to fuel kinetics at altitude.

The lower oxidation of carbohydrate at altitude is intriguing as the literature suggests that the metabolic energy pathways would favor optimizing the energy yield per unit of O_2_. This may only partially be explained by the significantly lower total energy expenditure at altitude (~11%) compared with sea level. This could have been expected, as even though the relative exercise intensities were matched based on altitude specific $\dot{V}O_{2\max}$_,_ the absolute workload at altitude (114.9 ± 9.7 watts) was lower than at sea level (132.6 ± 12.3 watts). This design was also adopted by Peronnet et al. (2006), as outlined above, who demonstrated contrasting findings. Therefore, the lower total energy expenditure at altitude is unlikely to be the full explanation, particularly as the magnitude of difference does not equate to the far greater absolute difference in energy expenditure from whole body carbohydrate oxidation (~45%).

The reduced whole body carbohydrate oxidation at altitude, particularly in the second hour of exercise, is explained by the reduced endogenous carbohydrate contribution to the total energy yield. Specifically, a reduced reliance on preexisting muscle glycogen stores as there were no real differences in the use of glucose released from the liver. These data are supported by the only other study to the authors knowledge to measure muscle glycogen use at altitude (Young et al. 1982), although not following carbohydrate ingestion. Young et al. (1982) showed a *large* (ES: 1.63) decrease in muscle glycogen use (10.9 mmol·kg^−1^) following acute altitude exposure (4300 m) compared with sea level. This was despite a lack of difference in whole body carbohydrate oxidation between sea level and acute altitude exposure.

In conjunction with the reduced reliance on preexisting muscle glycogen stores, free fatty acid concentrations were *moderately* higher at altitude compared with sea level in this study. Elevated free fatty acid concentrations by heparin administration have been shown to decrease muscle glycogen oxidation (Costill et al. 1977), which may explain these findings. Increased fat availability may increase NADH (reduced nicotinamide adenine dinucleotide) to mitigate against the potential fall in the cellular energy charge, downregulating pyruvate dehydrogenase (PDH) (pyruvate oxidation) via increased pyruvate dehydrogenase kinase (PDK) activity, reducing muscle glycogenolysis (Spriet and Watt 2003). Insulin was unchanged, which is also an important regulator of PDK activity and expression, with lower levels of insulin thought to be associated with reduced PDK inhibition (Peters et al. 2001). However, the downregulation of PDH may be potentiated at altitude by proliferator‐activated receptor (PPAR)α as discussed in more detail below. A limitation of this study is that we did not measure PDK and PDH activity or expression, as taking muscle biopsies was beyond the scope of this study. Therefore, further research is required to confirm the precise mechanism as well as why there was such variability in these muscle glycogen data compared with the other markers of fuel use.

The peak exogenous carbohydrate oxidation rates derived from glucose and fructose at sea level are comparable to the existing literature (Jentjens et al. 2004), demonstrating a significant contribution to the total energy yield. However, the rates of exogenous carbohydrate oxidation were slightly lower at altitude compared with sea level throughout the exercise period, only reaching statistical significance at 60 and 120 min, accompanied by *moderately* lower and higher plasma glucose concentrations, respectively. This is relevant as glucose oxidation is controlled in part by plasma glucose concentrations (Hawley et al. 1994). The lack of consistency in the plasma glucose concentrations being either lower or higher at altitude compared with sea level, does not support the phenomenon observed by Fulco et al. (2005) and Peronnet et al. (2006), who reported that plasma glucose concentrations following carbohydrate ingestion were higher during acute exposure to altitude. Nevertheless, the differences in rates of exogenous carbohydrate oxidation were not large enough for the relative or absolute contributions to the total energy yield to be significantly different between conditions. These data support the only other ^13^C tracer study at high altitude (Peronnet et al. 2006). However, in comparison the relative contributions of exogenous carbohydrate oxidation to the total energy yield were much greater in the present study compared with Peronnet et al. (2006) at both sea level (32.3 ± 3.1% vs. 12.5 ± 1.5%) and altitude (30.9 ± 3.7% vs. 14.9 ± 1.1%). Therefore, ingestion of a solution containing glucose and fructose (1.8 g·min^−1^) resulted in much higher exogenous carbohydrate oxidation during both conditions compared with glucose (1.75 g·min^−1^) alone (Peronnet et al. 2006), which is consistent with the sea level literature (~28% exogenous carbohydrate oxidation contribution (Jentjens et al. 2004)). The greater availability of carbohydrate in the present study is due to the likely higher overall carbohydrate absorption rates following the coingestion of glucose and fructose compared with glucose alone (Shi et al. 1995), due to their distinct intestinal transport mechanisms. Glucose uses the sodium‐dependent transporter (SGLT1) (Ferraris and Diamond 1997), which becomes saturated at a glucose ingestion rate of 1.0–1.2 g·min^−1^, and any further increase in glucose intake will not further increase exogenous glucose oxidation (Wagenmakers et al. 1993). In contrast, fructose is absorbed from the intestine by a separate mechanism, a sodium‐independent transport (GLUT5) (Wright et al. 2003). Therefore, there is less competition for absorption compared with isoenergtic amounts of glucose likely increasing the availability of carbohydrate for oxidation.

The relative contribution of fat oxidation to the total energy yield during the second hour of exercise at sea level (26.9 ± 13.1%) is similar to the existing literature (Smith et al. 2010; Peronnet et al. 2006; Jentjens and Jeukendrup 2005), taking into consideration the relative exercise intensity, dose of carbohydrate ingested, and the training status of the participants. However, a unique finding of the present study is the large differences in fat oxidation, with higher absolute (difference of 33.0 g for the 2 h test period) and relative contributions (difference of 24.3% during the second hour of exercise) to the total energy yield at altitude compared with sea level. In contrast, Peronnet et al. (2006) demonstrated a reduced reliance on fat oxidation (relative contribution of 8.0 ± 2.1%) at simulated altitude (4300 m in a hypobaric chamber) during the last 40 min of 80 min cycling at ~77% $\dot{V}O_{2\max}$ compared with sea level (21.9 ± 1.8%). These data are distinctly different, despite both studies matching the relative exercise intensity, which means a lower absolute workload during the hypoxic conditions. Whether this is due to the lower altitude and the coingestion of glucose and fructose in the present study compared with glucose alone needs to be established, as the relative exercise intensity in the present study was similar (~74% $\dot{V}O_{2\max}$).

The increased fat oxidation at altitude is supported by *moderately* higher free fatty acid concentrations in comparison to sea level during the exercise period. The higher free fatty acid concentrations at altitude are comparable to some studies (Jones et al. 1972; Roberts et al. 1996a; Fulco et al. 2005) and in contrast to other literature (Lundby and Van Hall 2002). However, the free fatty acid concentrations, with the *moderately* higher metanephrine and normetanephrine concentrations at altitude are likely to be indicative of greater fat lipolysis. It has been proposed that increased free fatty acid availability to the muscles will increase fat oxidation, as it leads to the downregulation of PDH (as already described) and phosphofructokinase (Spriet and Watt 2003)), both important regulators of glycolysis. There is no reason to suggest this mechanism would interact differently at altitude. Furthermore, the rate of free fatty acid utilization has been shown to be proportional to free fatty acid concentrations in animals (Paul 1970).

The increased fat oxidation could also be explained by the upregulation of the fatty acid‐activation transcription factor PPARα specifically in muscle fibers during hypoxia (Aragones et al. 2008). PPARα activation is a mechanism for a metabolic shift toward fat oxidation (Gilde and Van Bilsen 2003) and PPARα has also been shown to impair the activation of PDH via the upregulation of the PDK4 isoform (Aragones et al. 2008; Huang et al. 2002). This would limit the conversion of pyruvate to acetyl‐CoA in the skeletal muscle, hence reducing the synthesis of malonyl‐CoA, an inhibitor of fatty acid oxidation, thereby facilitating the oxidation of fat (Ruderman et al. 1999). Furthermore, there is also evidence from a rat study that beta oxidation in the liver is increased during acute hypoxia (4300 m) (Ni et al. 2015). Impaired entry of pyruvate into the TCA cycle is supported by the higher blood lactate concentrations at altitude in the present study, which likely reflects a greater rate of glycolysis but a reduced oxidative metabolism of carbohydrate. This fits the theory that hypoxia inducible factor 1‐alpha upregulates glycolysis at altitude, but within the present study it would seem that downregulation of PDH may play a more significant role. However, whether these mechanisms explain the findings of this study are yet to be confirmed, and further investigation is warranted.

In conclusion, acute exposure to hypoxia reduced the reliance on carbohydrate oxidation compared with normoxia during 2 h of cycling at the same relative exercise intensity, even in the presence of the abundant provision of multiple transportable carbohydrates. This finding is explained by a reduction in the use of preexisting muscle glycogen stores, rather than a real difference in the use of glucose released from the liver. Conversely, whole body fat oxidation increased, potentially explained by the *moderately* higher free fatty acid, metanephrine and normetanephrine concentrations. These findings might be explained by the downregulation of PDH in a hypoxic environment. However, this study did not measure metabolic signals to establish the mechanisms behind its unique findings, which should be a focus of future research.

## Conflict of Interest

The authors report no conflict of interest.
